# Cognitive abstraction increases prosociality when loyalty is valued lowly, but decreases prosociality when loyalty is valued highly

**DOI:** 10.1038/s41598-025-09158-w

**Published:** 2025-07-04

**Authors:** Gijs van Houwelingen, Marius van Dijke

**Affiliations:** 1 Netherlands Organization for Applied Scientific Research, The Hague, The Netherlands; 2 Department of Business-Society Management, Rotterdam School of Management, Erasmus University Rotterdam, P.O. Box 1738, 3000 DR Rotterdam, The Netherlands

**Keywords:** Charitable giving, Donations, Prosocial behavior, Cognitive abstraction, Loyalty, Impartiality construal level theory, Psychology, Human behaviour

## Abstract

Many studies show that people donate more to charitable causes that are presented in concrete (vs. abstract) terms; yet other research suggests that cognitive abstraction (vs. concreteness) encourages prosocial behavior. We propose that abstract cognition facilitates prosocial behavior among people who lowly value loyalty (i.e., those who value impartiality); concrete cognition should facilitate prosocial behaviors among people who highly value loyalty. Across three experiments and one cross-sectional survey in which we operationalize cognitive abstraction (vs. concreteness), valuing loyalty, and prosocial behavior in different ways, we consistently find that abstraction facilitates prosocial behaviors among people who lowly value loyalty. In two of the four studies, we also find that concreteness facilitates prosocial behavior among people who highly value loyalty. These findings help resolve theoretical ambiguity about the cognitive underpinnings of prosociality, and they have important practical implications for optimal framing of charity appeals to potential donors.

Are you more likely to donate money when asked to help children in need *or* when asked to provide poor children with breakfast every morning^1^? More generally, does abstraction or concreteness elicit prosocial behaviors? This is an important question. Practically, appeals for help or charitable donations can be phrased in both abstract (e.g., “help save the planet”) and concrete (e.g., “help save Keiko the orca”) terms. Theoretically, abstraction is a pervasive characteristic of people’s construal of any situation^2,3^. It may therefore help explain when and why people enact prosocial behaviors such as donating to charities, or fail to do so.

Research on the “identifiable victim effect” shows that people donate more to charitable causes described in concrete than in abstract terms. For instance, studies show that victims who are presented to potential donors with identifiable details (name, photo, background story) receive significantly higher donations than victims presented in abstract, statistical terms^4–6^. One influential explanation for this effect is that it is easier to feel empathy for identified than for abstractly described victims. For instance, research shows that a solitary identifiable victim triggers higher levels of empathy than the same individual as part of a larger group, and this leads to increased charitable giving^7^. Other research shows that viewing an identifiable victim activates brain regions associated with empathy, which predicts increased donations^8^.

In contrast with the findings on the identifiable victim effect, building on construal level theory, scholars have suggested that cognitive abstraction may lead to higher prosociality^9,10^. Cognitive abstraction (vs. concreteness) refers to mentally representing broad, inclusive classes of objects or events by reducing representation of the particular and idiosyncratic features of individual members of these classes^11,12^. Concrete (vs. abstract) cognition, on the other hand, is relatively contextualized and detailed^3^. Scholars have argued that, because abstraction (vs. concreteness) facilitates expansion of one’s mental horizons, it allows people to direct prosociality to a broader set of targets. The evidence for this conjecture is, however, indirect. Research shows that cognitive abstraction (vs. concreteness) predicts purchasing products for prosocial reasons (e.g., sustainability^13^), it makes justice being applied to a broader set of targets^14^, and it stimulates maximization of collective (vs. individual) outcomes^15^.

The aim of the present paper is to increase theoretical clarity about when cognitive abstraction, and when cognitive concreteness will lead to more prosocial behavior. Our reasoning starts with acknowledging that prosocial behavior is to an appreciable extent moral behavior^16–19^. We will argue that cognitive abstraction leads to more prosociality than concreteness among people for who impartiality is a guiding moral principle; on the other hand, cognitive concreteness should lead to more prosociality than abstractness among people for who partiality (i.e., loyalty) is a guiding moral principle.

Impartiality is a core element of utilitarianism. This moral philosophy implies that we are morally obliged to maximize the wellbeing of as many animate beings as possible such that each “is to count for one and none for more than one”^20^instead of privileging someone. People can apply utilitarian reasoning in their moral decisions. For instance, in the classic trolley dilemma, 70–80% of people attempt to maximize the wellbeing of five workers by sacrificing a single worker, although subtle changes to the situation can drastically decrease this percentage, such as making the agent harm the victim in a more direct manner than in the classic dilemma^21^. Utilitarian reasoning may also influence decisions to help others who are in need^22^. For instance, people who self-report that they support impartial concern for the well-being of everyone also self-report higher donations to charitable causes^23,24^.

As noted, Cognitive abstraction (vs. concreteness) refers to mentally representing broad, inclusive classes of objects or events by reducing representation of the particular and idiosyncratic features of individual members of these classes^11,12^. Abstract cognition can thus be characterized as a relatively non-differentiated form of cognition^3^: When people engage in cognitive abstraction, they tend to focus on communalities and to gloss over differences between targets. For example, abstract cognition has been associated with a less differentiated understanding of other people^25^. Based on these characteristics of cognitive abstractness (vs. concreteness), we expect it to lead to prosocial behavior rooted in the desire to impartially maximize the wellbeing of many.

The moral principle that is diametrically opposed to impartiality is loyalty. It is “a fundamental moral value” that makes people perceive and think “about people, groups, and organizations in ways that lead him or her to act on their behalf, even when doing so might come at a personal cost” and then “rationalize those actions as the “right thing to do””^26,27^. Loyalty has strong implications in collective settings. For instance, research shows that people respond more leniently to moral violations committed by people with whom they have a close relationship^28,29^ compared to violations committed by distant others, employees often respond negatively to whistleblowers who reveal unpleasant truths about their organization^30,31^people are willing to accept unethical request based on group loyalty^32^and they even support friends of people that they feel loyal to^33^. Applied to helping behavior, strongly valuing loyalty thus suggests helping others as a way to act upon their behalf, however indirect the connection may be.

As noted, concrete (vs. abstract) cognition is relatively contextualized and detailed, allowing to make fine-grained distinctions^3^. To illustrate, concreteness (vs. abstraction) lowers social stereotyping because it involves categorizing others as individuals rather than as members of social groups^23^. Helping out of a loyalty motive implies helping others as a way to act upon their behalf. For this reason, we expect that cognitive concreteness (vs. abstractness) leads to prosocial behavior rooted in strong endorsement of loyalty.

In the present paper we focus on chronic individual differences in the extent to which people value loyalty (vs. impartiality). Theories in moral psychology, such as the “Big Three of Morality”^14^moral foundations theory^18^social relations models^19^and the theory of ‘morality-as-cooperation^16^” view the strength with which people value loyalty as a variable that chronically varies between individuals and cultures. Empirical research supports the proposition that chronic individual differences in the strength with which people value loyalty (vs. impartiality) have meaningful effects. For instance, people who chronically value loyalty have been found to be more willing to help ingroup members^34^and to engage in costly forms of punishment for cheaters^35^. In sum, the above reasoning leads to the following hypothesis:

The strength of a person’s loyalty endorsement moderates the effect of cognitive abstraction on prosocial behavior such that, when loyalty endorsement is weak,* abstract (vs. concrete) cognition increases prosocial behavior*, whereas when loyalty endorsement is strong, abstract (vs. concrete) cognition decreases prosocial behavior (H1).

## Overview and transparency and openness statement

We conducted three experiments and one cross-sectional survey to test our hypothesis. All studies were approved by the Economics and Business Ethical Committee (EBEC) of the University of Amsterdam under number 20,181,203,041,221. We treated all participants in accordance with regulations of the American Psychological Association (APA). We obtained informed consent from all participants.

All measures we used were published in scientific journals which allow the use of the measures for scientific research. We describe our sampling plan, all data exclusions, all manipulations, and all measures. We made the data, research protocols and all items used in the studies available at https://osf.io/5nbm6/?view_only=e9bac179adbb490cb21c7b2e6af1af8b.

Sensitivity analysis using the powerPoisson function from the *powerMediation* package for R^36^ indicated that all studies had a power of 0.80 to detect at least a small-sized effect (d ≤ 0.2)^37^.

## Study 1

Study 1 was an experiment. Consistent with literature on the identifiable victim effect, we operationalized prosocial behavior as donations to a charitable cause. We manipulated cognitive abstraction by describing the cause either abstractly or concretely to participants. We measured individual differences in loyalty endorsement. We expected that participants who weakly endorse loyalty would donate more to the abstractly than concretely described cause, while participants who strongly endorse loyalty would donate more to the concretely than the abstractly described cause.

### Method

#### Design

We included goal abstraction (vs. concreteness) as a two-level between-subjects factor and loyalty as a continuous between-subjects predictor in our design.

#### Participants

We invited 300 UK or US participants through Prolific Academic. Of these, one failed to progress beyond the first page of the study, reducing our dataset to *N* = 299. Of the participants, 179 indicated that they identified as female, 119 as male, and 1 as another gender (*M*_age_ = 37.30, *SD* = 13.52).

#### Procedure and measures

We invited participants to what we described as a study on decision-making. We told participants that we would first like to know their opinion on “things that matter to them” and then would ask them to respond to various options. The first set of items was the 6-item MFQ subscale, which measures individual differences in the extent to which people value loyalty^18^. The loyalty scale consists of two subscales (relevance and judgment items). Relevance items are introduced with “When you decide whether something is right or wrong, to what extent are the following considerations relevant to your thinking?” (e.g., “Whether or not someone showed a lack of loyalty”). Respondents use a 7-point Likert scale (1 = “not at all relevant; this consideration has nothing to do with my judgments of right and wrong”; 7 = “extremely relevant; this is one of the most important factors when I judge right and wrong”) to respond. Judgment items are statements commonly related to loyalty (e.g., “I am proud of my country’s history”) to which respondents indicate their agreement using a 7-point Likert-scale (1 = “strongly disagree”; 7 = “strongly agree”). We averaged responses into an index of the value attached to loyalty (*M*_*abstract condition*_ = 3.67, *SD* = 0.81; *M*_*concrete condition*_ = 3.60, *SD* = 0.84, Cronbach’s α = 0.69).

After this, we introduced our measure of prosocial behavior. We told participants that they were eligible to receive a bonus of GBP 0.50 on top of their compensation for our study. We also informed them that they could keep this bonus for themselves or donate some or all of it to a charity.

At this point, we induced variations in different participants’ construal of the target of prosocial behavior (i.e., a charitable cause). In the concrete condition, the cause was presented in specific terms (i.e., “your donation will be used to serve breakfast, so kids don’t go to school hungry”). In the abstract condition, we provided a description in which such detail was absent (i.e., “your donation will be used to address hunger among kids”)^1^. See the online supplements for details.

We then asked participants how much of their bonus they donated. Their donation (in pennies) constituted our operationalization of prosocial behavior (*M*_*abstract condition*_ = 21.66, *SD* = 21.66; *M*_*concrete condition*_ = 23.87, *SD* = 21.69). After participants had made their donation, they were fully debriefed. We transferred the total sum donated to an account of United Way, a charitable organization. Finally, we collected demographic information and then thanked and fully debriefed participants.

## Results

Initial analyses revealed overdispersion in the data (*ratio* = 20.83, χ^2^ = 6122.80, *p* < .001). Therefore, we estimated the model using negative binomial regression (1 = *abstract*; -1 = *concrete*) with the glm.nb function from the *R* package MASS^38^. This model revealed significant main effects of goal abstraction (*b* = 0.10, *SE* = 0.02, *z* = 4.06, *p* < .001, 95% CI[0.05;0.14], *IRR* = 1.10), and of valuing loyalty (*b* = 0.06, *SE* = 0.02, *z* = 3.50, *p* < .001, 95% CI[0.02;0.10], *IRR* = 1.07). These main effects were qualified by a significant Goal Abstraction × Valuing Loyalty interaction effect (*b* = − 0.10, *SE* = 0.02, *z* = -3.96, *p* < .001, 95% CI[-0.15;-0.05], *IRR* = 0.91).

We proceeded to probe this interaction. Simple slopes analyses showed that at 1 *SD* above the mean on loyalty, abstraction (vs. concreteness) did not influence donations (*b* = 0.01, *z* = 0.26, *p* = .794); at 1 *SD* below the mean on loyalty, concreteness (vs. abstraction) significantly increased donations (*b* = 0.19, *z* = 5.47, *p* < .001).

However, a critical limitation of simple slopes tests is that the values that are chosen to test for simple slopes (usually 1 *SD* above and below the mean of the moderator) are arbitrary^39^. We therefore proceeded to use Johnson-Neyman (J-N) analyses: Rather than assuming that 1 *SD* above and below the mean are meaningful values of the moderator that should be contrasted with each other, the J-N method pinpoints the exact transition from non-significance to significance.

**Fig. 1:**
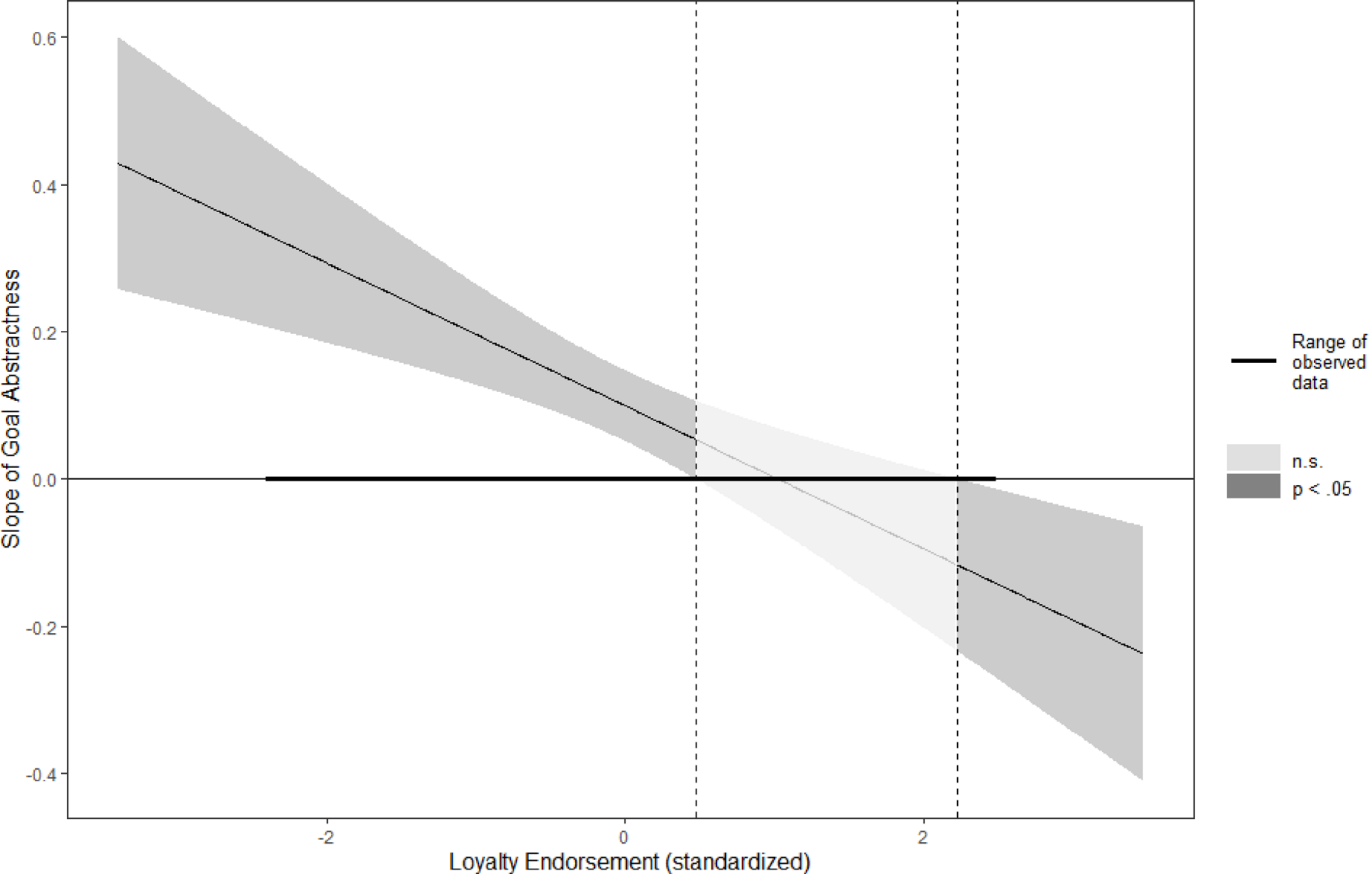
Regions of significance for the simple slope of goal abstractness on donations as a function of loyalty valuing in Study 1. The slope is significant outside of the two vertical dotted lines (i.e., in the dark-grey areas). Curved lines on each side of the slope represent 95% confidence intervals around the slope.

The J-N analyses revealed that an abstractly described charity received significantly (*z* > 1.96, *p* < .05) higher donations than a concretely described one from participants who weakly valued loyalty (i.e., from those scoring lower than 0.48 *SD* above the mean on valuing loyalty). A concretely described charity received significantly (*z* > 1.96, *p* < .05) higher donations than an abstractly described one from participants who strongly valued loyalty (i.e., from those scoring higher than 2.22 *SD* above the mean on valuing loyalty; Fig. 1).

### Discussion of study 1 and introduction to study 2

Study 1 offered first evidence consistent with our hypothesis. Yet, we obtained these results using one specific operationalization of prosocial behavior (donations to a charitable cause), and using one specific operationalization of cognitive abstraction, that is, goal abstractness. In our preregistered Study 2 we introduced diversity to the operationalizations of cognitive concreteness (vs. abstraction) and of prosocial behavior. We operationalized prosocial behavior as monetary support of a fellow participant and induced differences in cognitive abstraction using the established Category-Exemplar Task^15,35^.

## Study 2

### Method

#### Preregistration and transparency

Our analysis plan, including power analysis, target sample size, and exclusion criteria, are available on AsPredicted.org: aspredicted.org/NCC_P8V.

#### Design

We included cognitive concreteness (vs. abstraction) as a between-subjects factor in our design. We included valuing loyalty as a continuous between-subjects predictor.

#### Participants

We invited 250 UK or US based participants for our study on Prolific. Two failed to finish the study. We further excluded 11 participants from our final sample for failure of an instructed attention check item or who indicated that their responses were unreliable (or both) as per the conditions outlined in our preregistration, providing us with an effective *N* of 237, *M*_age_ = 40.19, *SD* = 13.12, 115 males (48.5%), 121 females (51.1%), 2 people (0.9%) who identified as another gender.

#### Procedure and measures

As in Study 1, participants first completed the 6-item loyalty subscale of the MFQ^15^ (*M*_*abstract condition*_ = 3.58, *SD* = 0.88; *M*_*concrete condition*_ = 3.48, *SD* = 0.83; Cronbach’s α = 0.68). Hereafter, we induced either abstract or concrete cognition using the Category-Exemplar Task^15,39^. In each condition, participants were given twenty-four prompts referring to common everyday objects (e.g., soda, book, movie; full list in the online supplement). In the abstract condition, we asked participants to provide descriptions of categories to which these prompts belong (e.g., beverage, medium, entertainment). In the concrete condition, we asked participants to provide descriptions of exemplars of the prompts (e.g., Fanta, Harry Potter V, the Avengers).

Then, we asked participants to make a decision that would affect both themselves and a fellow participant. They would divide 10 valuable “Experimental Currency Units” (“ECUs”) between themselves and the fellow participant. We indicated that they could think of ECUs as money (1 ECU = € 1,-). On average, participants gave 4.23 ECUs (*SD* = 1.62) to the fellow participant in the concrete condition and 3.84 ECUs (*SD* = 1.74) in the abstract condition.

Subsequently, we administered an attention check. We asked participants which of nine flavors of ice cream they preferred, but we instructed them to indicate “Smurf.” We filtered out those who choose a different flavor from our dataset. We also asked participants whether they thought their responses were reliable by asking them if they had been distracted while completing our study. After this, we collected demographic information and then thanked and debriefed participants.

## Results

**Fig. 2:**
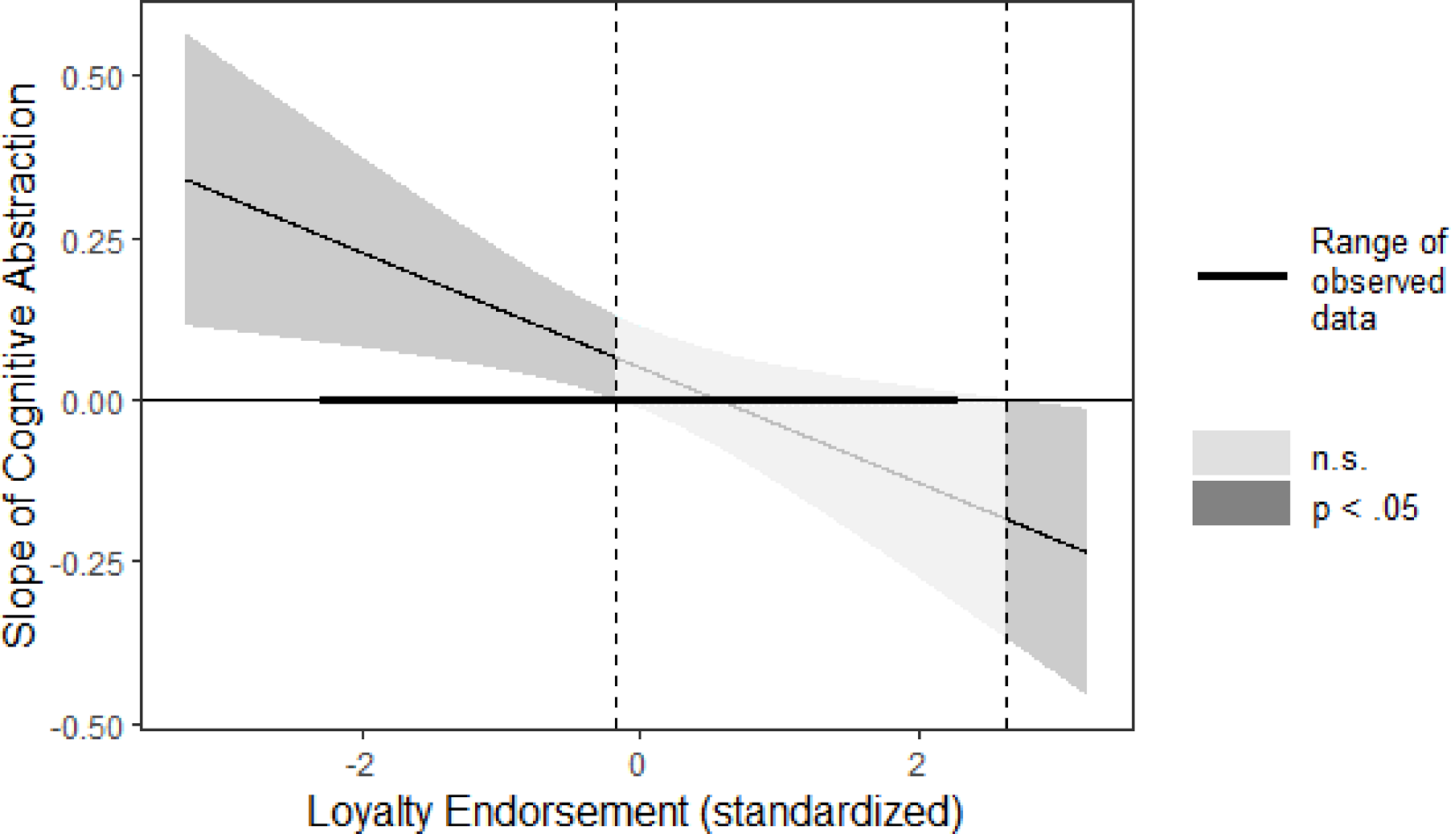
Regions of significance for the simple slope of goal abstractness on donations as a function of loyalty valuing in Study 2. The slope is significant in the left dark-grey area. Curved lines on each side of the slope represent 95% confidence intervals around the slope.

Initial analyses revealed no overdispersion in the data (*ratio* = 0.71, χ^2^ = 166.41, *p* = .99). Therefore, we estimated a Poisson regression model using the glm-function from base-*R* with prosocial behavior as the dependent variable and cognitive concreteness vs. abstraction (-1 = *concreteness*; 1 = *abstraction*), valuing loyalty and their interaction as predictors. We found no significant effect of valuing loyalty (*b* = 0.09, *SE* = 0.05, *z* = 1.86, *p* = .062, 95% CI[-0.004;0.19], and IRR = 1.09) or cognitive abstraction (*b* = 0.09, *SE* = 0.05, *z* = 1.53, *p* = .127, 95% CI[-0.03;0.23], and IRR = 1.10). The analysis also revealed a significant Cognitive Abstraction × Valuing Loyalty interaction effect (*b* = − 0.18, *SE* = 0.07, *z* = -2.65, *p* = .008, 95% CI[-0.31;-0.05], and IRR = 0.84; Fig. 2).

Simple slopes tests showed that at 1 *SD* below the mean on loyalty, abstraction (vs. concreteness) did not significantly influence ECUs given to the fellow participant (*b* = 0.18, *z* = 2.41, *p* = .016). At 1 *SD* above the mean on loyalty, concreteness (vs. abstraction) significantly did not increase ECUs given to the fellow participant (*b* = 0.05, *z* = 0.67, *p* = .502).

More appropriate J-N analyses revealed that cognitive abstraction led to significantly (*z* = 1.96, *p* < .05) more ECUs given to the fellow participant than concreteness by participants who lowly valued loyalty (i.e., those scoring lower than.18 *SD* above the mean. For participants who valued loyalty highly, cognitive abstraction led to less ECUs given to the fellow participant than concreteness, but this simple effect did not reach statistical significance within the observed range of the data.

### Discussion of study 2 and brief description of study 3

Study 2 revealed partial support for our hypothesis: abstraction (vs. concreteness) increased prosocial behavior among participants who lowly valued loyalty, but concreteness (vs. abstraction) did not increase prosocial behavior among those who highly value loyalty. We used an established cognitive abstraction induction (which was different from the one in Study 1) and a different measure of prosocial behavior than in Study 1.

In Study 3, we measured individual differences in cognitive abstraction (vs. concreteness) with the Behavioral Identification Form (BIF)^40^. We measured prosocial behavior with the Circle of Moral Regard measure^41^. Finally, in Studies 1–2 we measured valuing loyalty immediately before inducing cognitive abstraction, which may have influenced responses to the subsequent cognitive abstraction induction. In Study 3 we randomized the order in which we presented the measures. Because Study 3 is a correlational study, we briefly describe it here and fully describe it in the online supplements.

Negative binomial regression revealed a significant Cognitive Abstraction × Valuing Loyalty interaction. In support of the hypothesis, J-N analyses showed that high (vs. low) cognitive abstraction significantly predicted increased prosocial behavior among participants who valued loyalty lowly; among participants who highly valued loyalty high (vs. low) cognitive abstraction predicted significantly decreased prosocial behavior.

### Discussion of study 3 and introduction to study 4

Study 3 supports our hypothesis, this time using chronic individual differences in cognitive abstraction (vs. concreteness) and yet another measure of prosocial behavior. Together, Studies 1–3 suggest that our findings generalize beyond specific operationalizations of cognitive abstraction and prosocial behavior.

However, Studies 1–3 all used the same measure of valuing loyalty. Furthermore, from Studies 1–2 it does not become clear what specifically drives the effect of the cognitive abstraction manipulations: Is it abstractness vs. concreteness, or is a moderate (in-between) position on the cognitive abstraction dimensions sufficient to produce a contrast with abstraction or concreteness? In Study 4, a replication of Study 2, we included a control condition to the construal level manipulation. Second, we used a different scale to measure the value that people attach to loyalty than in Studies 1–3.

## Study 4

### Method

#### Design

We included cognitive abstraction as a three-level between-subjects factor in our design (abstract vs. concrete vs. control). We included valuing loyalty as continuous between-subjects predictor.

#### Participants

We invited 300 UK or US based participants through Prolific. Of the 300 participants, 167 (53%) identified as male, 130 (44%) identified as female, and 2 (0.7%) identified as “another gender. The mean age was 34.37 years (*SD* = 11.71).

#### Procedure and measures

We invited participants for “a study about social decision making”. We excluded participants in Studies 1–2 from participating. We first administered the scale to measure the value that people attach to loyalty that we developed. The items were: (1) betrayal is always immoral, (2) when a friendship or a relationship hits a rocky patch, it is your duty to remain faithful, (3) people should not let down the groups that they belong to, (4) betraying friends is wrong, (5) one should always avoid being duplicitous, and (6) whatever the circumstances, it is important to follow through on your promises (1 = “strongly disagree”; 7 = “strongly agree”; *M*_*abstract condition*_ = 3.97, *SD* = 0.53; *M*_*concrete condition*_ = 3.90, *SD* = 0.61; *M*_*control condition*_ = 3.90, *SD* = 0.51, Cronbach’s α = 0.68). We describe the scale validation in the online supplement.

Then, we administered the Category – Exemplar Task, as in Study 2. The only difference was that, in addition to the abstract and concrete condition we added a control condition, where participants were asked to write down their first association with the given prompt.

Finally, we measured *prosocial behavior* using the same dictator game as in Study 2. The number of valuable points donated to the other participant constituted the dependent variable (*M*_*abstract condition*_ = 4.17, *SD* = 1.55; *M*_*concrete condition*_ = 3.62, *SD* = 1.81; *M*_*control condition*_ = 4.05, *SD* = 1.66).

## Results

Initial analyses revealed overdispersion in the data. We therefore estimated a negative binomial regression. In this model, we found the predicted interaction effect of Valuing Loyalty × Abstraction (vs. Concreteness) on donations, b = − 0.29, SE = 0.29, *z* = -2.21, *p* = .027, 95%CI[-0.54;-0.03]. We did not find significant Valuing Loyalty × Abstraction (vs. Control) effect, *b* = − 0.13, SE = 0.14, *z* = − 0.97, *p* = .333, 95%CI[-0.42;0.11] or Valuing Loyalty × Concreteness (vs. Control), *b* = − 0.15, SE = 0.13, *z* = -1.15, *p* = .252, 95%CI[-0.40;0.13], interaction effects. In other words, the effects we observed in Studies 1–3 studies are driven by abstract vs. concrete cognition, rather than by abstract cognition vs. a type of cognition that is in-between abstract and concrete, or by concrete cognition vs. a type of cognition that is in-between abstract and concrete.

Simple slopes analyses showed that at 1 *SD* below the mean on loyalty, abstraction (vs. concreteness) significantly increased donations (*b* = 0.30, *z* = 2.88, *p* = .011). At 1 *SD* above the mean on loyalty, concreteness (vs. abstraction) did not significantly increase donations (*b* = 0.01, *z* = 0.09, *p* = .996).

**Fig. 3:**
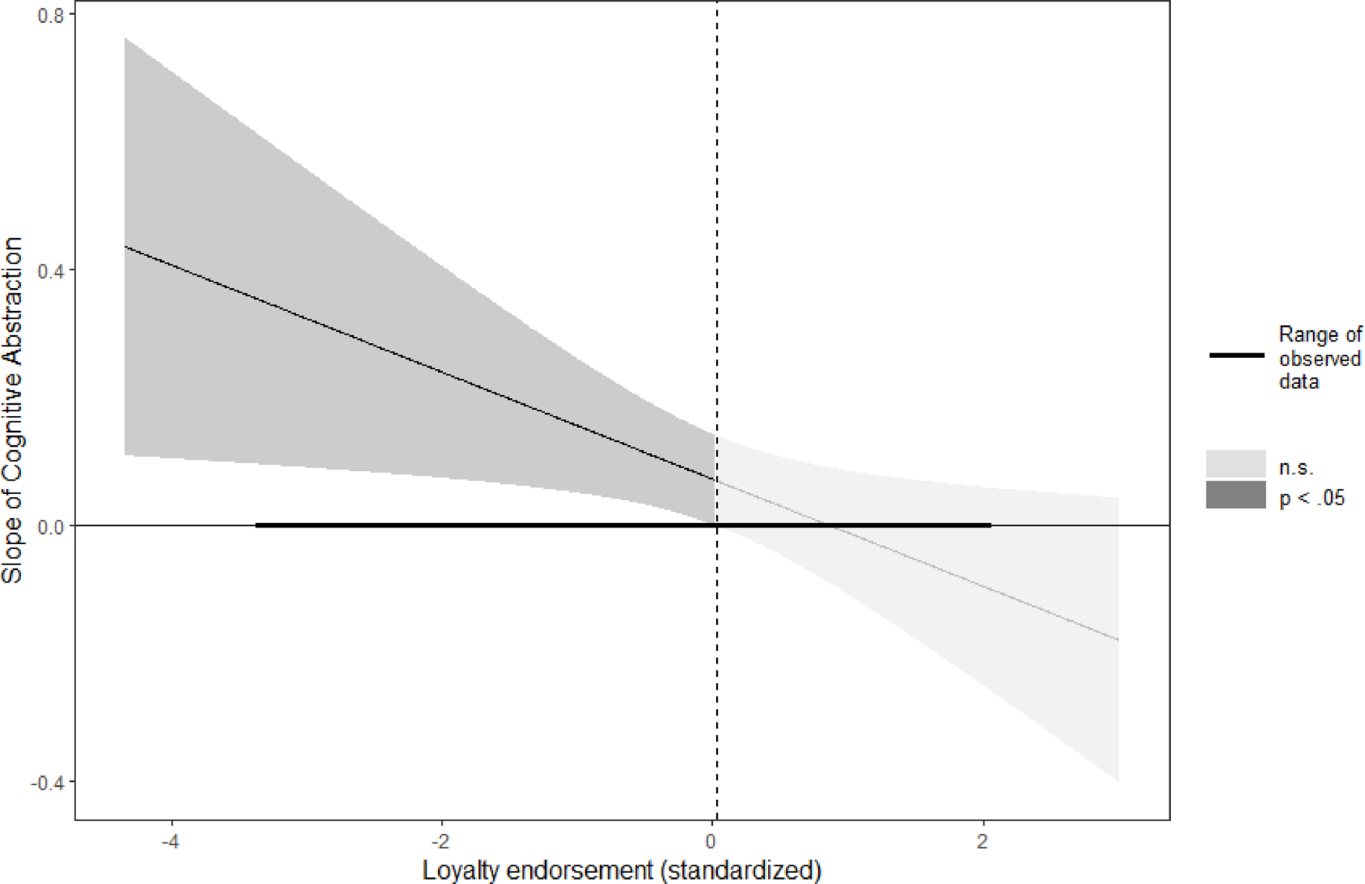
Regions of significance for the simple slope of goal abstractness on donations as a function of loyalty valuing in Study 4. The slope is significant in the left dark-grey area. Curved lines on each side of the slope represent 95% confidence intervals around the slope.

J-N analyses showed that abstractness led to more donations than concreteness (*z* > 1.96, *p* < .05) among participants who lowly valued loyalty (those scoring lower than 0.25 *SD* above the mean on valuing loyalty). Among participants who valued loyalty highly (1.96 *SD* above the mean on valuing loyalty, abstractness (vs. concreteness) decreased donations, although this effect was not statistically significant. *b* = − 0.28, *SE* = 0.25, *t* = -1.13, *p* = .130, 95%CI[-0.70;0.13] (Fig. 3).

## General discussion

In four studies, we found that among individuals who value loyalty lowly, cognitive abstraction (vs. concreteness) predicts or causes increased prosocial behavior. The other part of our hypothesis, that among individuals who value loyalty highly, cognitive concreteness (vs. abstraction) would lead to increased prosocial behavior was supported in Study 1 and 3 but not in Study 2 and 4. We tested our hypothesis by inducing variations in the abstractness of the goal of prosocial behavior (Study 1), by inducing variations in the abstractness of participants’ mindset (Studies 2, 4) and by measuring individual differences in abstraction (Study 3). We operationalized prosociality as donations aimed at charity (Study 1) or at a fellow participant (Studies 2, 4), and as prosocial intentions towards many different targets (Study 3). Finally, we measured the value people attach to loyalty with the established MFQ subscale (Studies 1–3) and with a newly developed scale (Study 4).

### Implications

Construal level scholars have speculated that cognitive abstraction (vs. concreteness) may stimulate prosocial behavior^9,10^. However, only a few studies provide indirect support for this idea^13–15^. Our research provides the first explicit account of when cognitive abstraction promotes, and when it undermines prosocial behavior. Abstract cognition facilitates prosocial behavior among people for who impartiality is a moral imperative (i.e., those who lowly value loyalty); we also found some evidence that cognitive concreteness facilitates prosocial behavior among people who for who partiality as a moral imperative (i.e., people who highly value loyalty).

Our results also have implications for the literature on the identifiable victim effect. It has been shown that vivid displays of a single person in need increase donations because such appeals trigger empathy towards the victim^7,8^. Our findings from Studies 1 and 3 suggest that the identifiable victim effect is limited to people for whom partiality is a moral imperative, and reverses among people who focus on impartiality. In so doing, we help identifying mechanisms that limit, and even reverse the identifiable victim effect.

Our research is relevant to individuals or organizations that aim to stimulate people to donate to charitable causes, such as organizations that engage in nonprofit marketing or cause-marketing but also governments, and even supervisors within organizations who want to stimulate prosocial behavior among employees. Our results make clear that there is no “one best way” to stimulate people to donate (e.g., by communicating relatively abstractly or relatively concretely). Rather, different people can be encouraged to donate through different communicative strategies. Those who strongly value loyalty may respond more positively to a concrete appeal, while those who weakly value loyalty may be more readily persuaded to engage in prosocial purchases or donations by framing a message in abstract terms.

Returning to our opening example, our findings suggest that political conservatives (who relatively strongly value loyalty)^18^ may be more willing to donate to help provide poor children with breakfast every morning, whereas liberals (who value loyalty less strongly) may be more willing to donate money when asked to help children in need^1^.

Finally, individuals or organizations who seek to encourage prosociality should consider whether their message makes salient the value of loyalty among their audience. Simply thinking about loyalty may induce this concept and may do so independently from chronic individual differences in how strongly people value loyalty^24^. Messages that, for instance portray victorious sports teams, people sacrificing themselves for relatives or their country, or cohesive groups of friends, may make the value of loyalty salient, and this may make target audiences contribute more to concretely than abstractly described causes.

### Limitations and suggestions for future research

We measured the value that people attach to loyalty directly before inducing cognitive abstraction (vs. concreteness) in Studies 1, 2 and 4. Although this approach ensures that the cognitive abstraction manipulation did not influence the measurement of loyalty, it could alter participants’ responses to the manipulation. However, in Study 3, where we measured our concepts of interest in randomized order, we found the same interaction pattern as in Studies 1, 2 and 4. We thus have no evidence that the procedure we used in our experiments biased our results.

Our measure of prosocial behavior in Studies 1, 2 and 4 (i.e., structured as a dictator game^42^) strongly resembles behaviors such as charitable giving. They thus mirror measures that are typically used in the identifiable victim literature. Future research should also include charitable behaviors such as donating time or blood. These outcomes structurally fit the dictator game, but they may be experienced differently by participants (Macdonnell & White, 2015). Future research should also test if the effects of cognitive abstraction (as a function of valuing loyalty) materialize in close relationships, where loyalty is especially valued^25,26,43^ and where prosocial behaviors such as providing emotional support may be more prominent. Here, we note that our effect of interest generalizes to the number of targets for whom people feel personally responsible for their wellbeing (i.e., prosocial inclusion; Study 3).

Cognitive concreteness (vs. abstraction) did not facilitate prosociality among participants who highly value loyalty in Studies 2 and 4, where we directly induced cognitive concreteness (vs. abstraction), but it did in Study 1, where we operationalized the target of prosociality in concrete vs. abstract terms (as in identifiable victim studies) and in Study 3, where we measured individual differences in cognitive abstraction. Future research should test if cognitive concreteness (vs. abstraction) mediates the identifiable victim effect.

Finally, our evidence was obtained in short-lived, anonymous settings based on responses from participants living in the UK or the US. Although this mirrors many real-life donation settings, future studies should strive to include more culturally diverse participants to ensure that the conclusions drawn are robust and applicable on a global scale^44^.

### Concluding remarks

In showing that cognitive concreteness leads to prosocial behaviors among people who highly value loyalty while abstractness leads to prosocial behavior among people who lowly value loyalty (i.e., those driven by impartiality), our research helps create theoretical clarity about the cognitive underpinnings of prosociality and provides clear practical implications for optimal framing of charity appeals to potential donors.

## Data Availability

The data, the materials used in the studies and supplemental materials in which we report additional analyses are available at: https://osf.io/5nbm6/?view_only=e9bac179adbb490cb21c7b2e6af1af8b.
